# Annokey: an annotation tool based on key term search of the NCBI Entrez Gene database

**DOI:** 10.1186/1751-0473-9-15

**Published:** 2014-06-26

**Authors:** Daniel J Park, Tú Nguyen-Dumont, Sori Kang, Karin Verspoor, Bernard J Pope

**Affiliations:** 1 Genetic Epidemiology Laboratory, Department of Pathology, Medical Building, The University of Melbourne, Melbourne, Victoria 3010, Australia; 2 Department of Computing and Information Systems, Doug McDonell Building, The University of Melbourne, Melbourne, Victoria 3010, Australia; 3 Victorian Life Sciences Computation Initiative, The University of Melbourne, 187 Grattan Street, Melbourne, Victoria 3010, Australia

**Keywords:** Gene annotation, Keyword search, NCBI gene database, PubMed article summaries

## Abstract

**Background:**

The NCBI Entrez Gene and PubMed databases contain a wealth of high-quality information about genes for many different organisms. The NCBI Entrez online web-search interface is convenient for simple manual search for a small number of genes but impractical for the kinds of outputs seen in typical genomics projects.

**Results:**

We have developed an efficient open source tool implemented in Python called Annokey, which annotates gene lists with the results of a keyword search of the NCBI Entrez Gene database and linked Pubmed article information. The user steers the search by specifying a ranked list of keywords (including multi-word phrases and regular expressions) that are correlated with their topic of interest. Rank information of matched terms allows the user to guide further investigation.

We applied Annokey to the entire human Entrez Gene database using the key-term “DNA repair” and assessed its performance in identifying the 176 members of a published “gold standard” list of genes established to be involved in this pathway. For this test case we observed a sensitivity and specificity of 97% and 96%, respectively.

**Conclusions:**

Annokey facilitates the identification of genes related to an area of interest, a task which can be onerous if performed manually on a large number of genes. Annokey provides a way to capitalize on the high quality information provided by the Entrez Gene database allowing both scalability and compatibility with automated analysis pipelines, thus offering the potential to significantly enhance research productivity.

## Background

Recent advances in high-throughput DNA sequencing have allowed large regions of a sample genome (or genomes) to be sequenced rapidly at high coverage [1]. The detection of rare variant associations with disease is one of many applications enabled by this new technology [2]. However, current analysis pipelines tend to produce large numbers of genetic variants, only a small subset of which are likely to be relevant to the phenotype under study [3,4]. This necessitates filtering and annotation to obtain manageable lists of candidates for further investigation. A typical workflow includes the identification of a list of variants in a sample, annotation of those variants with metadata drawn from a variety of sources, and filtering using research-specific criteria. Existing annotation tools, such as Annovar [5], are good at associating variants with basic biological information, such as which genes are affected. However, they are less helpful in deciding whether those genes are relevant to a given domain of interest. For a single DNA sample it is not uncommon for an experiment to produce thousands of variants, which may be collectively associated with hundreds of genes. It is therefore infeasible to manually inspect every candidate gene to determine if it is known to be associated with a particular domain of investigation. In order to address this problem we have implemented the Annokey tool described in this paper. We discuss its main features, how it is used and implemented, and analyse its performance on a simple test case relative to a published “gold standard” of DNA repair pathway genes [6].

Annokey’s inputs are a list of genes and a list of key terms (e.g. “DNA repair pathways”) that are considered by the user to be relevant to a research question. For each gene, Annokey searches for instances of those terms within the NCBI Entrez Gene database [7] and PubMed article abstracts and records their frequency and occurrence contexts. This provides an indication of each gene’s relevance as a candidate for follow-up study. The key terms are listed by the user in descending order of significance, and the rankings of matched terms are included in the output. This offers an additional level of information for prioritisation, which becomes more important as the number of key terms increases. The tool automates what would otherwise be a labour intensive task and can greatly increase research productivity. Whilst Annokey was originally developed in the context of rare variant detection, it is applicable to a much wider range of genomic studies; any situation where a researcher wants to know which of a set of genes are related to particular topics, e.g. RNA-Seq, ChIP-Seq, siRNA screens.

The Entrez Gene database is provided by the National Center for Biotechnology Information (NCBI) and contains detailed information about genomes from a variety of organisms. Gene records for a particular organism are curated by NCBI’s Reference Sequence project, drawing data from other databases within NCBI and other sources such as the Gene Ontology Database [8]. These represent individual genes and each is labelled by a unique identifier called a GeneID. Each entry contains information about (amongst other things) nomenclature, gene sequences, gene product sequences, pathways, interactions, markers and phenotypes. Entries also indicate other resources within and outside the NCBI, including literature citations in the form of PubMed article references. The database can be accessed in two main ways. The first access method is via a web interface [9] which is suitable for interactive browsing and querying. The second access method is via a programming interface (which is used by Annokey). Whilst the web interface provides an expressive search engine, it is tedious to use for large numbers of genes or large numbers of search terms, and it is difficult to incorporate into an automated analysis pipeline. The programming interface is also not straightforward to work with for non-technical researchers, as also noted by Mrozek *et al.* who aim to provide a more user-friendly interface for complex exploration of the NCBI resources [10]. Annokey is a targeted gene annotation tool that allows researchers to capitalize on the high quality information provided by the Entrez Gene database in a way that scales to large numbers of genes and queries, is tailored to a specified user context, and is compatible with automated analysis pipelines.

Annokey operates from the command line and has the following four main features:

1. Online and offline search capability.

2. Flexible search terms, allowing use of regular expressions. Search terms are ranked in importance by the user according to the order in which they are listed in the input.

3. Summary search results are provided as annotations on the input gene list for quick inspection, prioritisation and integration with workflows based on spreadsheets.

4. Detailed search results are provided in an HTML report with hyperlinks back to the Entrez Gene web interface. The HTML report also shows each matching instance of a search term and the context in which it was found.

Annokey’s online search retrieves data from the NCBI databases directly over the Internet. This provides access to the most recent version of the data but, due to network latencies, is typically only applicable to queries involving a small number of genes (<100), although the number of search terms is not a limiting factor. Offline search utilises a locally cached copy of the Entrez Gene database, which can be populated using a snapshot of the whole database for a given organism. Offline search is appropriate for queries involving hundreds or thousands of genes and search terms, or in cases where a large number of different searches are required. The Annokey distribution provides an additional tool that automates the process of downloading and preparing the latest database snapshot.

Search terms are provided by the user in a text file, one per line, and are written as regular expressions [11]. This provides an easy interface for exact-match literal terms but also allows sophisticated search patterns to be constructed. For example, the search term “tumor cell” matches exactly (and only) that text, whereas “([Cc]ancer|[Tt]umou?r) [Cc]ell” generalises the search to allow for both “cancer cell” or “tumor cell”, an optional spelling of “tumour” (with a “u”), and variation in capitalisation of the first letter of each word. The use of regular expressions provides a good compromise between the needs and abilities of novice and advanced users. The terms are ranked by importance according to the relative order of the lines in the input file. In its summary output, Annokey annotates each gene with information about the search results. One of the annotations is the highest rank of any matching search term, which provides a simple way for the user to prioritise the results.

For each gene in the input, Annokey retrieves its corresponding entry from the Entrez Gene database (if it exists), and then searches for each keyword in the fields of that entry. The location and frequency of each search term is recorded. The search optionally extends to the PubMed article summaries referenced in the gene record. The results of the search are presented in two ways: 1) as summary annotations to the input gene file; 2) as a more detailed HTML report. The summary output provides information that is easy to understand at a glance and practical to use in a typical bioinformatics workflow using spreadsheets (or CSV files). The detailed output provides a more fine-grained breakdown of the search results and includes hyperlinks back to the Entrez Gene web interface. We anticipate that most users will first look at the summary results to select a set of candidate genes, and then use the detailed report to investigate the candidates in more depth.

### Implementation

Annokey is implemented in Python 2.7, is intended to be used as a command-line application, and can be used on any POSIX-compatible operating system (e.g. Linux, Mac OSX).

The algorithm is realised by the ANNOKEY_SEARCH procedure, which takes four parameters: 1) a list of gene names; 2) a list of search terms; 3) an organism name (which defaults to human); and 4) a Boolean flag indicating whether the search should include PubMed articles referenced from the gene entries. To avoid ambiguities such as those caused by synonyms, Annokey matches input gene names against the “Official Symbol” as specified by NCBI.

Each gene is processed separately by the loop spanning lines 4 to 26. The database record for a gene is retrieved (line 6) as an XML document, and parsed into its constituent fields (line 7). The precise set of fields that are searched are presented in the “NCBI Entrez Gene database fields” subsection below. Depending on how Annokey is used, the gene record might be found in the local file cache, or it might be fetched by an online query to the NCBI Entrez database. Each field of the gene record is processed separately by the loop spanning lines 9 to 13, and for a given field, each search term is processed separately by the loop spanning lines 11 to 13. All the matches of the search term are collected (line 12) and added to the set of hits (line 13). Each hit records the gene name, the search term, the field in which the matches were found, and a list of match locations. If the parameter “include_pubmed” is true then Annokey will also search through the titles and summaries of PubMed articles that are referred to in each gene record (lines 15 to 26). The set of PubMed articles referenced by a gene are collected (line 17) and the corresponding PubMed database entries are retrieved (line 18). Annokey first looks for PubMed database entries in a local file cache, and if that is unsuccessful, it then tries to fetch them from the online PubMed database (saving the downloaded entry into the local cache to optimise future requests). The XML PubMed entries are parsed into fields and searched in much the same way as the fields of the gene database. The total set of search hits is returned on line 28. In practice, to save memory, Annokey incrementally generates outputs after each gene is processed. It further reduces memory requirements by employing a streaming XML parser [12], which avoids the need to store the entire entry in memory for any given gene. Multiple different genes in a search could refer to the same PubMed article. Annokey avoids repeating the same search by saving the results of previous searches in a table indexed by PubMed ID.

Annokey uses the BioPython [13] library to request data from the online Entrez Gene and PubMed databases, and optimises the retrieval database entries by keeping a local file cache of database records. Cached retrieval of database entries avoids the latency of network access, which significantly reduces the running time of the program, and also reduces the load on the NCBI servers. Snapshots of the Entrez Gene database are provided periodically on the NCBI FTP server [14]. Annokey provides an additional tool to automate the process of populating its local file cache from a database snapshot. At the time of writing the uncompressed snapshot for human genes was roughly twelve gigabytes, with similar sizes for other organisms. Annokey’s local cache stores each gene entry in a separate XML file, using a directory structure indexed by organism and then by gene name. To reduce the load on the file system, the gene names are further distributed evenly over 256 sub-directories by hashing the gene name (with a stable hash function) modulo 256. During the development of Annokey we experimented with storing the file cache in a relational database but found a slight degradation in performance and so we opted for a simpler solution based on the filesystem. The PubMed database is much larger than Entrez Gene and also more tightly restricted in its terms of use. This means that Annokey cannot populate a local cache of PubMed from a snapshot in the same way that it does for Entrez Gene. However, Annokey does keep a cache of previously downloaded PubMed entries to avoid requesting the same entry more than once from the online servers. In future work we will consider using the downloadable snapshots of MEDLINE [15] the bibliographic database underlying the PubMed retrieval interface, to populate the PubMed cache.

The detailed HTML report is facilitated by the use of the “html” Python library [16] using version 5 of the HTML syntax, which is supported by all modern web browsers. The “hide/show details” facility in the report is implemented using embedded Javascript. We have validated the HTML report using the W3C validator [17].

### Results and discussion

#### Example usage

The following example illustrates how Annokey can be used to perform a simple search. More complex scenarios are described in the user documentation [18]. Annokey requires two input files: a list of genes, and a list of search terms. The input gene list is a comma or tab separated file with at least one column headed “Gene”. Other columns are allowed; they will be ignored by the search but preserved in the output. For illustrative purposes, suppose we have supplied the following gene list of three genes (although typically the list would be much longer, and contain many more columns):

Gene

RPA1

BRCA1

CANT1

The input search term list is a text file with one term per line. Search terms can be literal text or regular expressions. The example below illustrates the contents of a file with two terms (again, in a real experiment, the list would be much longer):

[Bb]reast [Cc]ancer

DNA [Rr]epair

If, for example, the gene list and the search term list are stored in files called “genes.csv” and “terms.txt” respectively, then an offline (cached) search can be performed by executing the following on the command line:

annokey --terms terms.txt --genes genes.csv

The summary output of the search is a new CSV file (printed to the standard output device) with three additional columns added, as follows:

Gene,Highest Rank,Highest Rank Term,Total Matched Entries

RPA1,2,DNA [Rr]epair,10

BRCA1,1,[Bb]reast [Cc]ancer,392

CANT1,,,

The output contains the original gene column (and any other columns in the input gene file) plus three additional columns of annotations, based on matches to the provided search terms:

1. Highest Rank: the numerical rank of the highest ranked matching search term.

2. Highest Rank Term: the highest ranked matching search term.

3. Total Matched Entries: for each search term Annokey counts the number of database fields where that term is found at least once. This column contains the sum of all those counts over all the search terms.

The first two annotation columns show the highest ranked matching term (its numerical rank, and the term itself). The numerical rank allows the user to sort the search results in terms of the relative importance of matched terms. In the example above, we have ranked “breast cancer” as having higher importance than “DNA repair”. The third annotation column shows how many matching fields were found over all the search terms. This allows the user to sort the results in terms of the weight of evidence. In the example above, there were no matches for the *CANT1* gene, so its annotations are empty. It is important to note that the annotated output provides a heavily summarised view of the search results. It is intended to provide an overview that is relatively easy to understand and prioritise. Brief, sortable summaries are particularly useful when dealing with large numbers (>100) genes, as is typical in many genomics projects. Annokey also provides more detailed search results in the form of a HTML report, which we describe below.

**Figure 1:**
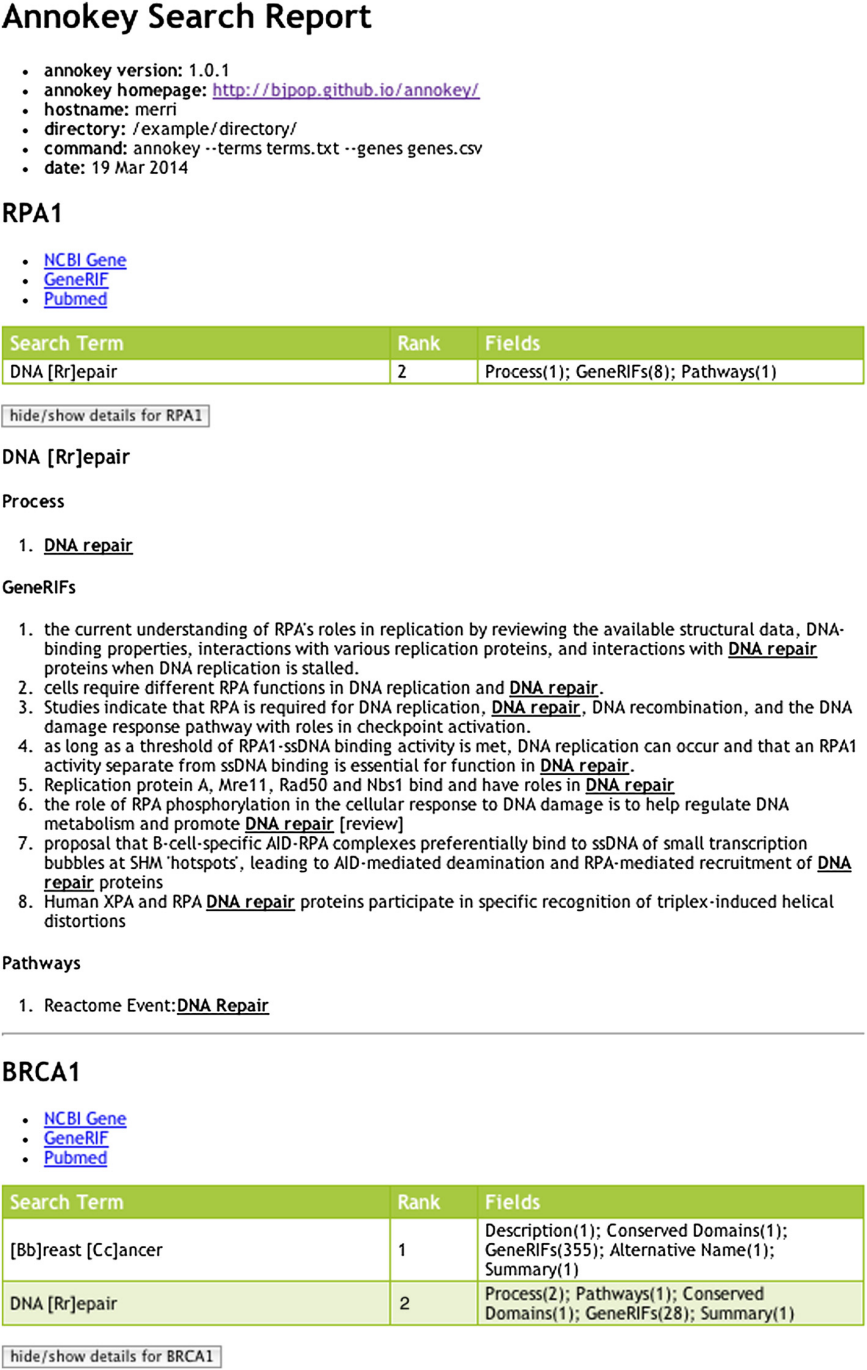
**Screen-shot of an example detailed search report, showing matches for two genes *RPA1 *and *BRCA1 *against the search terms “[Bb]reast [Cc]ancer” and “DNA [Rr]epair”.** The top part of the report shows meta information about the search. This is followed by an entry for each of the genes. Each gene entry contains hyperlinks back to the online Entrez Gene database entry for the gene, its list of GeneRIFs and PubMed references. The green coloured table summarises the matches for each search term, including the rank of the term and the fields where matches were found and their frequency. Following the table is a list of each search term showing all the contexts where the term was matched. This section can be quite long, so it is hidden by default, and can be expanded by clicking on the “hide/show details” button. In the picture above, the details are shown for *RPA1* but hidden for *BRCA1*.

The detailed output of the search is an HTML file containing an entry for each input gene describing the location and frequency of each matching term. Figure 1 shows the output for the example search above, as rendered by a web browser. The report begins with meta-information about the search, such as the version of Annokey that was used, the command line arguments and so forth. Then the search results are broken down by gene, and within a gene, by search term. The report only shows genes having at least one match, which explains the absence of *CANT1* in the example. Each gene entry contains a table showing, for each matching search term, its rank and the locations and frequencies of its matches in the various fields of the gene database. In the figure we can see that “DNA [Rr]epair” was ranked second and matched once in the Process and Pathways fields and eight times in the GeneRIF (Gene Reference into Function) fields. Following the table is a more detailed view showing the context of each search term match, where the term is highlighted within the field in which it was found. This section can become quite large, so it is hidden by default, and can be displayed by clicking on the “hide/show details” button for the corresponding gene.

### Analysis of performance

In order to gauge the effectiveness of Annokey, we measured its sensitivity and specificity against a published “gold standard” list of human DNA repair genes [6]. This is by no means an exhaustive experiment but it gives us an idea about its behaviour in a reasonably well-controlled scenario. We ran Annokey with an input list of all the human genes contained in the Entrez Gene database, searched for the key term “DNA [Rr]epair”, and included linked PubMed articles in the search. We counted every gene with at least one search-term match as a positive result (and any other gene as a negative result), and compared the list of positives against the gold standard. Any positive result that was also in the gold standard list was considered a “true positive”, whereas the remaining ones were considered “false positives”. Any gene in the gold standard list that was not also within the positive results was considered a “false negative”, whereas the remaining ones were considered “true negatives”. The results were as follows:

Number of human genes in Entrez Gene (at the time of the experiment): 43869

“True positives”: 170

“False positives”: 1543

“True negatives”: 42150

“False negatives”: 6

Sensitivity: 170/(170 + 6) = 0.97

Specificity: 42150/(42150 + 1543) = 0.96

We also examined “true negatives” and “false positives” by mixing a random sample of 100 genes from each set, and compared them to a blinded classification by a human expert. For each gene the expert manually looked up its entry on the Entrez Gene database and scored its relevance to “DNA repair” on a scale of 0 to 2, where 0 means not related, 1 means moderately related, and 2 means highly related, based on the evidence in the database (and not prior knowledge). The rules for scoring were defined before the experiment, and are listed in Table 1. The human expert agreed with Annokey on all 100 of the “true negatives” (all the “true negatives” were scored 0 by the human expert). In Annokey’s “false positive” set, the human expert classified 2 genes as moderately related and 16 genes as highly related, which is to say that the expert agreed with Annokey that 18/100 of the “false positives” with respect to the gold standard were in fact correctly classified. We expect a relatively high false positive rate with keyword search, especially in the situation where even a single match is considered to be a positive result. In practice a user is likely to apply more stringent thresholds and thus reduce the false positive rate (though perhaps at the expense of an increased false negative rate). We also believe that the high false positive rate of keyword search can be further mitigated by the ability of the user to rank the importance of the results in the summary output. Of course the question of “truth” is a moving target in genomics and our tests are limited to the information contained in the Entrez Gene database. However, we believe the experiment shows that keyword search of Entrez Gene is a simple and effective way to classify genes.

**Table 1:** Rules employed by a human expert to score the relevance of genes against the topic “DNA repair” using manual inspection of evidence from the Entrez Gene database

**Score**	**Definition**
2	Is most likely relevant to DNA repair or DNA damage response, supported by biochemical evidence of involvement in DNA damage response affecting DNA repair.
1	Is possibly involved in DNA repair or DNA damage response e.g., DNA repair protein binding partners without necessarily evidence of involvement in DNA repair, altered regulation in response to DNA damage or DNA damaging treatments but without direct evidence of a role in DNA repair.
0	Has no discernable involvement in DNA repair or DNA damage response and the key term match appears off-target e.g., refers to another gene mentioned in the same text as the test gene.

### Related work

Myriad tools exist to support functional analysis of gene lists. A 2009 survey identified nearly 70 tools that highlight “interesting” genes derived from high-throughput studies [19]. Many such tools take advantage of structured information in gene databases, including Gene Ontology annotations [8]. PubMed is used by some tools as a source of functional information about genes. However, these tools generally rely on statistical tests to establish the relevance of specific genes and their annotations with respect to a set of reference or control genes. In contrast, Annokey applies a user-directed ranking algorithm that relies on curated associations between genes and an area of interest.

There are also a number of tools that provide search functionality for the biomedical literature. Web-based, general search tools comparable to the PubMed system are reviewed in [20]. A few such tools offer the ability to limit results to particular biological entity types, such as genes, which can help to identify the literature most relevant to a given gene. A small number of literature search tools are also specifically designed to support the analysis of lists of genes. The most directly comparable in aims to Annokey are GoGene [21] and GeneValorization [22].

GoGene is a tool that draws on associations between genes and functionally-related terms extracted from the literature, in combination with structured information in Entrez Gene and UniProt, to organise genes according to particular functional categories (e.g. Gene Ontology terms). Genes related to specific functional categories or diseases can be identified by browsing the relevant ontology structures. Like Annokey (when the PubMed search is activated), it performs a search against both PubMed and Entrez Gene. Unlike Annokey, the direct literature search is based only on matches to the gene names in the abstracts. Associations between genes and specific diseases or functional concepts are based on identification of those concepts in the abstracts mentioning the genes. The tools are therefore somewhat complementary; while Annokey relies on direct links to the literature to identify relevant abstracts rather than the occurrence of a gene name, it can support more flexible search for relevant terms (including in the Gene database itself, not restricted to terms found in the literature alone) and users have direct control over the relative importance of those terms.

GeneValorization is a web-based tool that shows the relationship between genes and contexts of study (keywords) using frequency of co-location of the gene name and the keyword in the literature. It employs a graphical format to display its results. Whilst having similar goals to Annokey, it differs functionally in a number of ways. Annokey is a command-line tool that is designed to work with existing analysis pipelines, whereas GeneValorization is designed for interactive use. Annokey allows more flexible search using regular expressions, whereas GeneValorization uses literal terms only. Annokey searches within various fields of the Entrez Gene databases plus linked PubMed articles, whereas GeneValorization focuses on literature search only. Annokey caches database entries, which allows it to support very large numbers of genes and terms, whereas GeneValorization performs all queries online.

## Conclusions

Annokey is a freely available open-source tool that allows users to annotate a list of genes using a keyword search of the Entrez Gene database and linked PubMed article summaries. It produces two types of search results: 1) a summarised output of annotated genes, which is useful for filtering and prioritisation; and 2) a detailed search report which shows how each search term was matched within the various fields of a gene record. We believe that Annokey will be a useful addition to many bioinformatics workflows in which lists of candidate genes need to be prioritised with respect to a domain of interest.

### Availability and requirements

**Project name:** Annokey

**Project home page:**http://bjpop.github.io/annokey/

**Operating systems:** POSIX-like operating systems (OS X, Linux)

**Programming language:** Python

**Other requirements: Python libraries:** BioPython, lxml, html

**License:** BSD

**Any restrictions to use by non-academic:** None

## Abbreviations

NCBI: National Center for Biotechnology Information; CSV: Comma separated value; HTML: Hypertext markup language; XML: Extensible markup language.

## Competing interests

The authors declare that they have no competing interests.

## Authors’ contributions

DP, SK and BP contributed to the design and implementation of Annokey. DP, TN-D, KV and BP contributed to the analysis of Annokey’s sensitivity and specificity. All authors contributed to the drafting of the manuscript, and have read and approved the final version.
